# The Impact of Ions Contained in Concrete Pore Solutions on Natural Zeolites

**DOI:** 10.3390/ma16041416

**Published:** 2023-02-08

**Authors:** Przemysław Czapik

**Affiliations:** Faculty of Civil Engineering and Architecture, Kielce University of Technology, Al. Tysiąclecia Państwa Polskiego 7, 25-314 Kielce, Poland; p.czapik@tu.kielce.pl

**Keywords:** SEM, thermal analysis, X-ray diffraction, pozzolanic reaction, zeolites

## Abstract

This article investigates the relationships between different chemical compositions of simulated cement concrete pore solutions and changes on the surface of zeolite rock with potassium clinoptilolite as its main component. The changes were studied using X-ray diffraction (XRD), thermal analysis (DTA-TG) and scanning electron microscopy (SEM). Zeolite powder samples and a ground section of 16–64 mm grain were tested. The simulated pore solutions were based on Ca, Na, K hydroxides and K_2_SO_4_. It was found that 100% of Ca(OH)_2_ in the systems could react between 7 and 180 days of hydration due to pozzolanic and side reactions. As the degree of clinoptilolite conversion increased, it became more difficult to detect it in X-ray patterns. At the same time, various microstructural changes could be observed. As a result of the reactions that occurred, hydrated calcium silicates, sulfate and carbonate compounds were formed. Potassium hydroxide had a more substantial effect on clinoptilolite reactivity than sodium hydroxide. This effect can be enhanced by the presence of SO_2_^3−^ ions in the solution.

## 1. Introduction

Zeolites are tecto-aluminosilicates with characteristic framework structures enclosing a system of channels and cavities responsible for their high nanoporosities and specific surface areas [1,2]. The channels, 0.3 to 0.9 nm in size, allow atoms and small molecules to penetrate and diffuse into the internal structures [2], giving zeolites characteristic physical and chemical properties. As a result, zeolites are versatile in their industrial applications. An important practical application is the use of zeolites as sorbents [1]. In cement and concrete technologies, they are utilized as mineral raw materials for cement production and as mineral additives [3,4,5,6,7,8]. The use of zeolites as mineral additives is related to the acidic properties of zeolites, i.e., to the Brønsted and Lewis acid sites on their surfaces [9]. Their presence is determined by, among other things, the chemical composition of the mineral, the amount and type of exchangeable cations, and the concentration of structural defects. The Brnøsted acid sites, the main source of acidity in zeolites [2,9], allow them to react with alkaline solutions, which breaks the ionic bonds of the zeolite structure. If this happens in the presence of Ca^2+^ ions, the zeolite undergoes a pozzolanic reaction [1,2,4,6,9]. Zeolites in the C-A-S-H system can accompany the C-S-H phase in a wide range of compositions, especially silica-rich ones. However, in the C-S-H phase, zeolites are unstable when a significant amount of Ca(OH)_2_ is present [1,9,10]. Therefore, natural zeolites can be classified as pozzolans [10,11]. Such materials do not harden when mixed with water, but in finely ground form in the presence of water at normal ambient temperature they react with dissolved calcium hydroxide to form calcium silicate and calcium aluminate compounds of increased strength. Due to this property, zeolites are used in technologies in the fields of binding materials, autoclaved materials and concrete. Besides the C-S-H phase, pozzolanic reactions can also produce hydrated calcium aluminates C_4_AH_x_ (x = 9–13), gehlenite (C_2_ASH_8_), calcium carboaluminate (C_3_A·CaCO_3_·12H_2_O), ettringite (C_3_A·CaCO_3_·32H_2_O) and monosulfate (C_3_A·CaSO_3_·12H_2_O) [11].

The pozzolanic properties of zeolites also help mitigate alkali–silica reactions (ASRs) in concrete [4,5,12,13,14]. In this context, however, zeolites can have a variety of effects. Some products of alkali–silica reactions can crystallize from alkali–silica gel in the form of zeolites [15,16,17,18]. Zeolites contained in aggregates can be considered reactive phases that damage concrete by reacting with alkalis [17]. Similar to other siliceous materials used in cement and concrete manufacturing, the differences between zeolites used as pozzolanic additives to prevent ASR-induced concrete degradation and aggregates that cause such degradation are determined by their fineness [6,12,17,19,20,21]. These opposing zeolite effects in concrete are attributed to a similar mechanism. In a pozzolanic reaction, silica ions can react with Ca^2+^ to form a C-S-H phase and with Na^+^ and K^+^ to form an expansive silica gel.

The most common zeolite in nature, clinoptilolite, has such an ability. It is a heulandite-series mineral characterized by a high Si/Al ratio > 4.5 and a preference for Na^+^ and K^+^ as exchangeable cations [1,2]. In a strongly alkaline environment, it is unstable even at low temperatures. Depending on the starting composition and ambient temperature, it can transform into, for example, analcime, alkali feldspar, NaP zeolite, phillipsite, mordenite and faujasite [1]. Zeolites of the clinoptilolite group have also been detected as ASR products [15,16].

For the above reasons, clinoptilolite is the most commonly studied zeolite used as a mineral additive/binder. However, research tends to focus on its influence on the characteristics of a finished product, e.g., mortar/concrete [4,6]. Some research on the chemical properties of zeolites has been carried out with the aim of modifying zeolites to better improve building materials [2,22]. Still, the literature lacks a comprehensive study of the zeolite properties that determine their impact on the properties of concrete. Due to its pozzolanic properties, clinoptilolite was examined for the effect of calcium ions [23,24,25]. Note that calcium cations do not occur alone in concrete pore solutions [10,26,27,28] and that they are not the only cations that can react with clinoptilolite. Sodium and potassium cations are also present in the solutions and can trigger alkali–silica reactions, which, as already mentioned, are similar to pozzolanic reactions [23,29]. Nevertheless, the alkali reactivity of zeolitic rocks is rarely studied.

For this reason, the present study investigated how the most common ions present in pore solutions can affect zeolites containing clinoptilolite as their main component. This research aims to further characterize the chemical processes that zeolites present in concrete can undergo, thereby creating a basis for planning and interpreting future research.

In addition, the effect of SO_2_^3−^ on these processes was investigated, since reactive sodium and potassium in Portland cement clinker are normally present in the form of sulfates [10].

## 2. Materials and Methods

### 2.1. Test Methods

In order to precisely identify the material that is the subject of this study, namely, zeolite, tests were carried out to determine its grain size, composition and microstructure. This information supplemented the data supplied by the manufacturer. Some of these methods were then used to determine the effects of simulated pore solutions on zeolite.

Tests to determine the grain size of powdered zeolite, on which its specific surface and thus its reaction surface depend, were carried out on a Helos KR laser diffraction analyzer (Sympatec GmbH, Clausthal, Germany). The test results were given as the averages of three samples.

The mineral composition of the zeolite was characterized by analysing thin sections prepared from large zeolite pieces using transmitted light microscopy (OLYMPUS BX51—Olympus, Tokyo, Japan) and the point-counting technique. Both crossed polarizers and a single polarizer were used in the tests.

X-ray diffraction (XRD) was used to analyze the phase composition of the zeolite and its subsequent transitions due to the interactions with the simulated pore solutions. A PANalytical Empyrean diffractometer (PANalytical, Almelo, The Netherlands) with a Cu anode was used for this purpose. The scans covered an angular range of 5 to 70°2θ, using a step size of 3.3° min^−1^. The ICDD PDF-2 [30] database was used as a reference database for the diffractogram analysis. It was assumed that with constant measurement parameters and the same fineness, the peak intensity would only depend on the type and content of a specific phase in the sample.

During investigations of zeolite transformations due to the interactions of ions in the pore fluids, thermal analysis was applied to estimate the phase compositions and quantify the transformations occurring in the samples. Thermal analysis was performed on samples collected from the XRD samples. The TGA/DSC Q600 device (TA Instruments, New Castle, DE, USA) was used. The samples were heated from 50 to 1000 °C at a rate of 10 K/min.

The microstructure of the zeolite and the pore-solution ion effects were examined using scanning electron microscopy (SEM). A Quanta FEG 250 microscope obtained from the FEI Company (Hillsboro, OR, USA) equipped with a secondary electron detector (SE) and an energy-dispersive X-ray microanalyzer (EDS) were used. Before being placed under the microscope, the wet samples were allowed to dry under natural conditions for two hours without further sputtering to prevent any influence on their composition. The test was performed under a low vacuum (30 Pa) using an electron beam of 20 kV for image acquisition with the SE detector and an increased beam voltage of 30 kV for elemental analysis with the EDS detector.

### 2.2. Materials

#### 2.2.1. Zeolite

The object of the investigation described in this work was Transcarpathian zeolite from Western Ukraine [6,14]. In the remainder of this article, “zeolite” will refer to the zeolite rock as a whole and not merely to a single mineral within the zeolite group (clinoptilolite).

**Figure 1:**
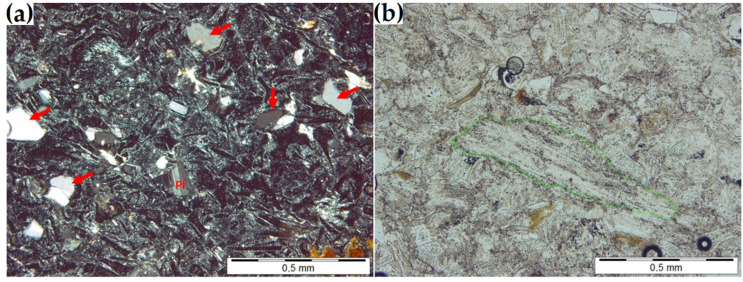
(**a**) Splinters (arrows) and polysynthetic twins within a plagioclase grain (Pl) (observed between two crossed polarizers). (**b**) Elongated pumice clast (marked in green) in the clinoptilolite mass (a single polarizer).

Table 1 shows the chemical composition of the zeolite. A petrographic examination was used to determine its mineral composition. The zeolite has a glass-shard-like structure typical of pyroclastic rock. Numerous fragments of volcanic glass, pumice and quartz, feldspar, and biotite crystals are distributed within the microcrystalline background composed of clinoptilolite and clay minerals (if present) (Figure 1). Table 2 summarizes the mineral composition of the zeolite sample determined with the planimetric method. The sample was determined petrographically to be zeolitic vitro-crystalloclastic tuff.

**Figure 2:**
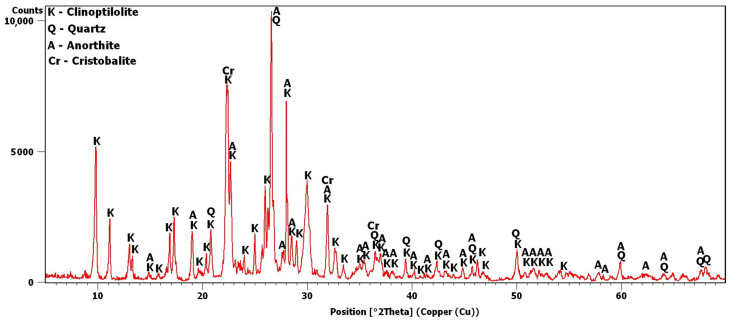
Transcarpathian zeolite X-ray diffraction pattern.

The potassium clinoptilolite content in this material, as reported by the manufacturer, based on XRD estimates, was 84%. Added to this was quartz, in an amount of 3%, and feldspar, in an amount of 6%. In this study, the X-ray analysis was repeated to obtain the diffraction pattern shown in Figure 2. Analysis of the pattern confirmed the presence of quartz and anorthite, i.e., plagioclase—calcium feldspar. The most intense peaks were observed around 9.8 and 22.4°2θ. The peak at 9.8°2θ was the most intense peak obtained for clinoptilolite [30]. The 22.4°2θ peak was more intense due to an additional phase, i.e., cristobalite, which is contained in volcanic glass [30,31].

Non-modified zeolite powder and large zeolite particles (16–64 mm) were used in the tests. Thin sections and ground-section samples were prepared.

**Figure 3:**
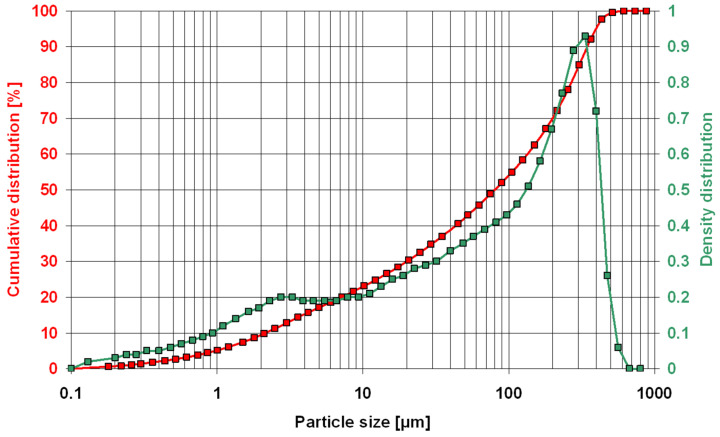
Particle size of the zeolite powder.

**Figure 4:**
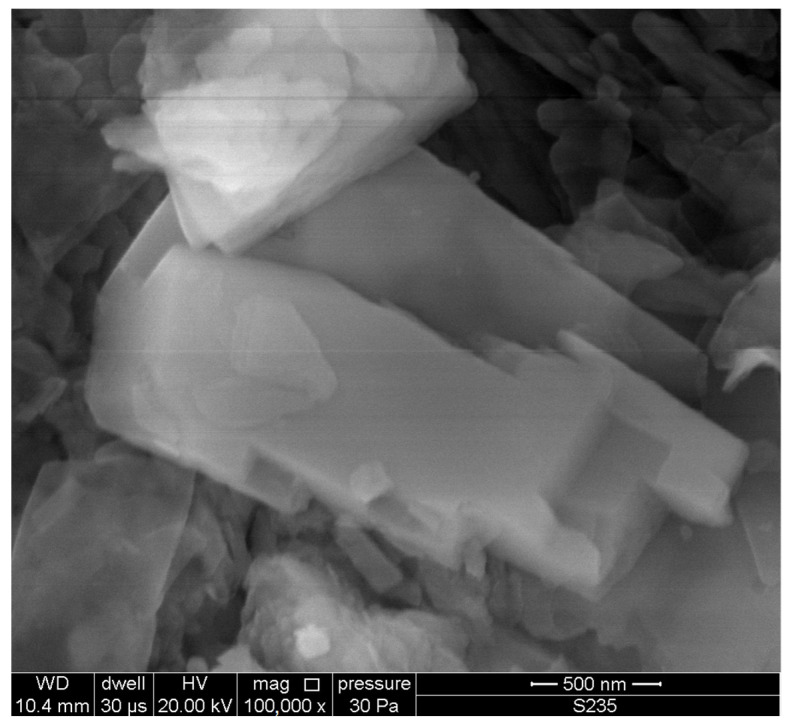
Clinoptilolite crystal in Transcarpathian zeolite.

The particle size of the zeolite powder was 0–0.35 mm, as determined by laser diffraction (Figure 3). Observations of the powder microstructure revealed the presence of numerous clinoptilolite crystals (Figure 4).

#### 2.2.2. Chemical Reagents

The solutions used to react with the zeolite were prepared using analytically pure Ca(OH)_2,_ NaOH, KOH and K_2_SO_4_ (Avantor, Gliwice, Poland). The simulated pore solutions were prepared with deionized water.

### 2.3. Procedures for Chemical Activity Tests

#### 2.3.1. Testing Powder Samples

The investigation of the chemical activity of zeolite in contact with different solutions containing Ca^2+^, Na^+^, K^+^ and SO_2_^3−^ ions involved the monitoring of phase transitions. The testing was based on the assumptions made for the pozzolanic activity tests [24]. For this, the zeolite powder was placed in alkaline solutions with compositions simulating the pore solution in concrete. Six samples with compositions as detailed in Table 3 were prepared.

The samples were placed in closed plastic containers. Material for XRD characterization was collected after 1, 3, 7, 14, 21, 28 and 180 days. Particular attention was paid to the changes that occurred in the peaks of Ca(OH)_2_ and clinoptilolite. After testing, the test material was discarded.

Following the X-ray examination, the samples collected after 7 and 180 days were also examined by thermal analysis (DTA-TG) to determine changes in the content of water bound in hydration products and Ca(OH)_2_ [32].

The microstructures of the 21-day samples were examined by scanning electron microscopy.

#### 2.3.2. Ground-Section Test

**Figure 5:**
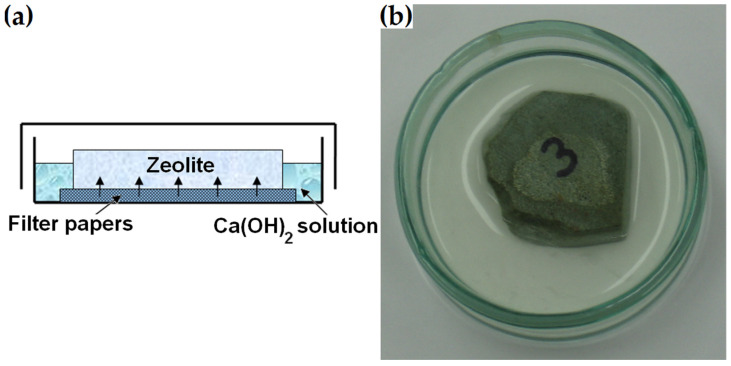
(**a**) Immersed ground section. (**b**) Photograph of the sample prepared for determining surface changes due to pozzolanic reactions.

The ground section was made from the zeolite piece previously used to prepare thin sections for mineral analysis described in Section 2.2.1. Unlike the polished-section preparation, the 16–64 mm zeolite piece was ground without further polishing or impregnation. Similar to the procedure described by Ortega et al. in [33], the bottom surface of the specimen was placed on filter paper in a glass container filled with a supersaturated calcium hydroxide solution prepared by mixing 5 mL of water and 5 g of Ca(OH)_2_ (Figure 5). After 1, 7, 14 and 21 days, the bottom surface of the dry ground section was analysed with respect to its microstructure under a scanning electron microscope. After four hours of microscopic examination, it was returned to the solution. The same regions of the sample were examined each time.

## 3. Results

### 3.1. Chemical Activity of Zeolite Powder

**Figure 6:**
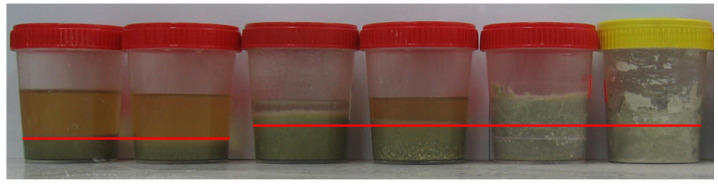
Twenty-eight-day zeolite powder samples; from the left: Z_n_, Z_k_, Z_c_, Z_cn_, Z_ck_ and Z_cks_.

Even before commencing the test, various effects of the applied alkaline solutions on the powdered zeolite could be observed. The solid phases of some samples increased in volume. Figure 6 shows a photo of 28-day samples. The red lines mark the original levels of the solid phases. The highest swelling potentials were observed in the Z_ck_ and Z_cks_ samples. The increases in the solid phases were accompanied by the disappearance of separate liquid phases present above the powder materials tested. After 21 days of testing, the separated liquid phases in Z_ck_ and Z_cks_ were negligible, but the samples remained wet throughout the 180-day test period. Storing the originally powdered samples Z_ck_ and Z_cks_ in the solution produced compacted materials of very low strength. Their strengths were so low that they could not stop the needle of the Vicat apparatus for testing cement [10], so the setting time could not be measured.

**Figure 7:**
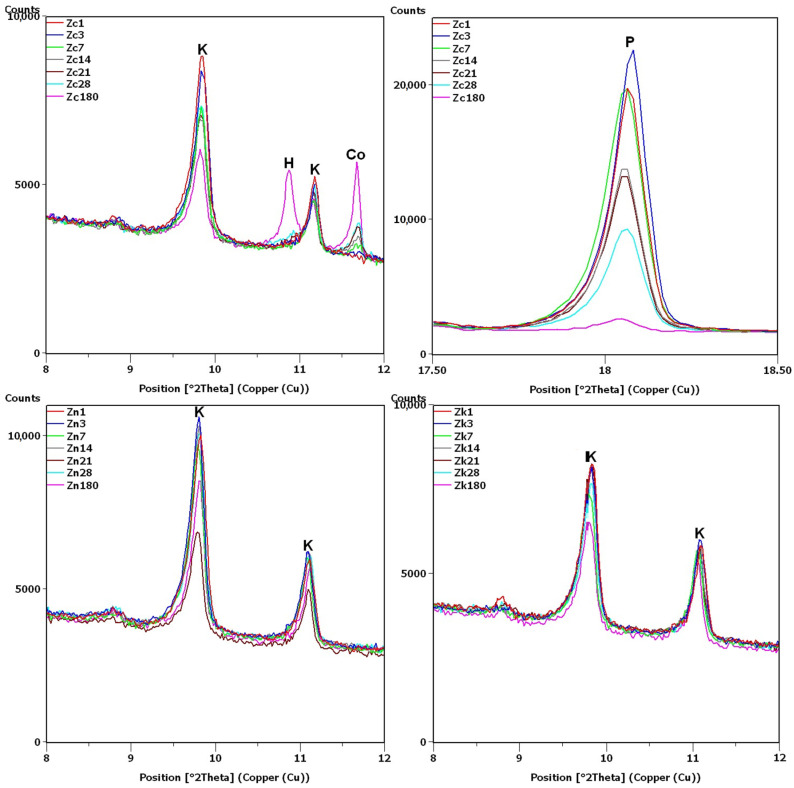
X-ray patterns of Z_c_, Z_n_ and Z_k_ samples in the areas of peaks characteristic of clinoptilolite (K), cowlesite (Co), hydrated calcium silicate (H) and portlandite (P).

**Figure 8:**
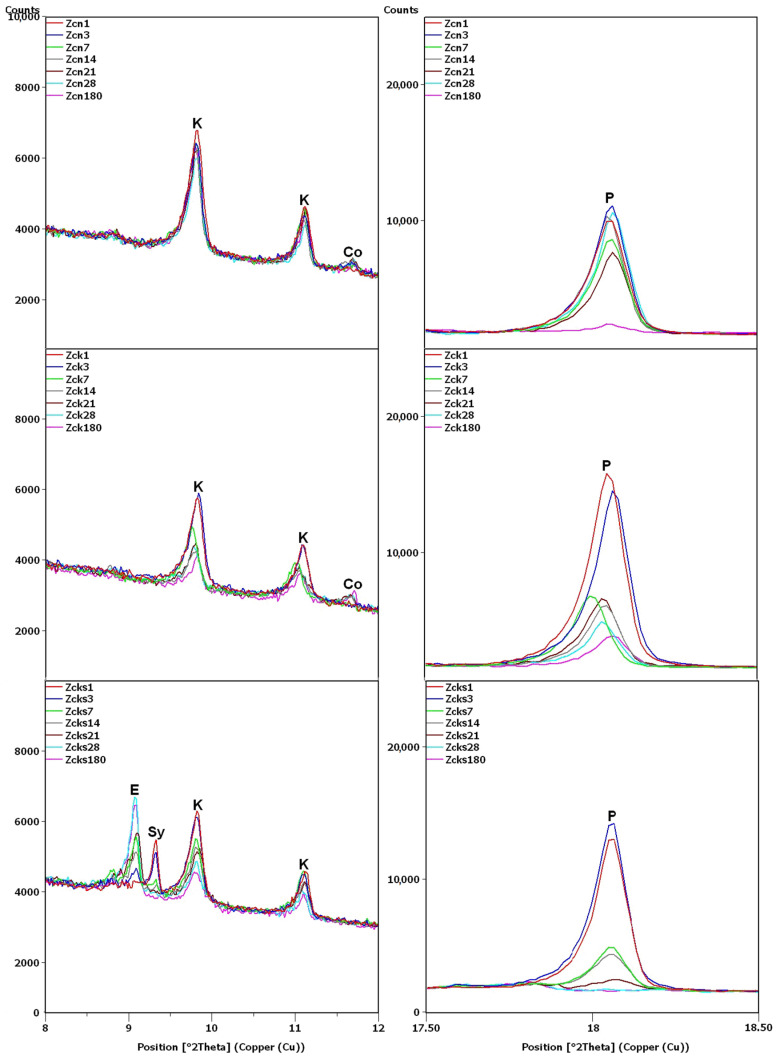
X-ray patterns of Z_cn_, Z_ck_ and Z_cks_ samples in the areas of peaks characteristic of clinoptilolite (K), ettringite (E), syngenite (Sy), cowlesite (Co) and portlandite (P).

**Figure 9:**
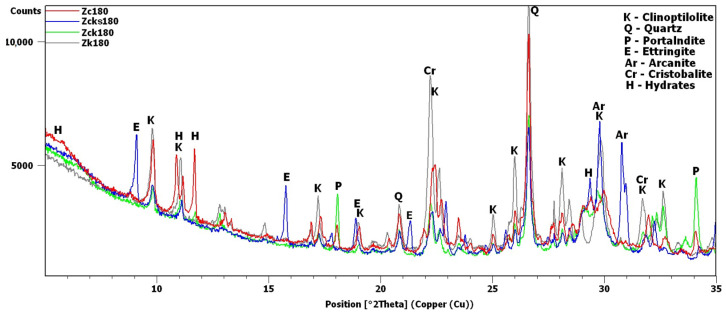
X-ray patterns of 180-day samples: Z_c_, Z_cks_, Z_ck_ and Z_k_.

XRD testing found the main differences between the samples, namely, the different intensities of the portlandite and clinoptilolite peaks. For this reason, the analysis of the diffraction patterns (Figure 7 and Figure 8) focused on the areas of peaks characteristic of these phases (8–12 2θ for clinoptilolite and 17.5–18.5 2θ for portlandite). Since no calcium hydroxide was present in the Z_n_ and Z_k_ samples, the second of these areas (portlandite) is not shown. In the description (Figure 7, Figure 8 and Figure 9) of the illustrated diffraction patterns, the number next to the sample name label indicates how many days after its preparation the test was performed. For samples using a single hydroxide solution (Z_n_, Z_k_ and Z_c_), significant changes in phase composition were observed mainly for the solution containing Ca^2+^ cations (Figure 7). The portlandite and clinoptilolite peaks decreased in intensity. It was also observed that the intensity of the clinoptilolite peaks decreased in samples without Ca(OH)_2_, although this change progressed more slowly. This observation was similar to that of Martinez-Ramirez et al. [25], except the portlandite must have carbonized in their study, since calcite was detected instead [34].

In the remaining Ca(OH)_2_-containing samples (Figure 8), the clinoptilolite and portlandite peaks were less intense than in the Z_c_ sample, with the presence of Na^+^ and K^+^ ions having different effects. In the Z_cn_ sample, the decrease in the intensity of the clinoptilolite peak was small, despite a significant reduction in the intensity of the portlandite peaks. In the Z_ck_ sample, the decrease in the clinoptilolite peak intensity was more pronounced than in the Z_c_ and Z_cn_ samples, but the portlandite peak intensity decreased more slowly than in the Z_cn_ sample.

The largest decrease in peak intensity for both clinoptilolite and portlandite was observed in the Z_cks_ sample. Portlandite was not detected in this sample after 28 days. In addition, ettringite was found to have formed in this sample, with a transient syngenite appearance.

Additional peaks were recorded for the Z_c_ sample from day 7, most likely due to the formation of hydrated calcium silicates with cowlesite-like structures. The peaks, which could be cowlesite peaks that appeared after a long time, were also detected in the Z_cn_ and Z_ck_ samples. Intense peaks related to hydrates, C-S-H phase [30,35], were also identified in sample Z_cks_ after 180 days (Figure 9).

After 180 days, the hydrate peaks were accompanied by increased background in the 2θ range of 5–7°, also present in the Z_ck_ and Z_cks_ samples at the time but at lower intensities (Figure 9). Figure 9 plots the increase in background against the Z_k_ sample, for which no increase was recorded. Comparison of Figure 2 and Figure 9 shows an evident decrease in the intensity of the common peak for clinoptilolite and cristobalite at 22.4°2θ for samples Z_c_, Z_ck_ and Z_cks_.

In the Z_cks_ sample (Figure 9), the extremely high clinoptilolite peak at 29.9°2θ can be explained by its overlap with the peak of arcanite (K_2_SO_4_), which crystallized from the solution used. The presence of arcanite was confirmed by the peak at angle 30.9°2θ.

The results of the thermal analysis are shown in Table 4. Four thermal effects were found. The first effect (maximum: 60–150 °C) was associated with the evaporation of water adsorbed on the surface of the grains and bound in the formed hydrates. Another effect (435–455 °C) was linked to the dehydroxylation of portlandite. The third effect (640–715 °C) was due to the decarbonation of carbonates from portlandite [34]. In the Z_cks_ sample examined after 7 days, an additional fourth effect (290 °C) occurred, which was related to the decomposition of syngenite [36]. These peaks corresponded to the mass losses recorded on the TG curves. The amount of water contained in the hydrates increased with time for each of the samples tested. The presence of portlandite and calcium carbonate was not observed in the Z_n_ and Z_k_ samples, as Ca(OH)_2_ was not added. These phases were detected in the remaining samples, but their levels tended to decrease significantly over time. The content of carbonates increased slightly only in the Z_c_ sample.

**Figure 10:**
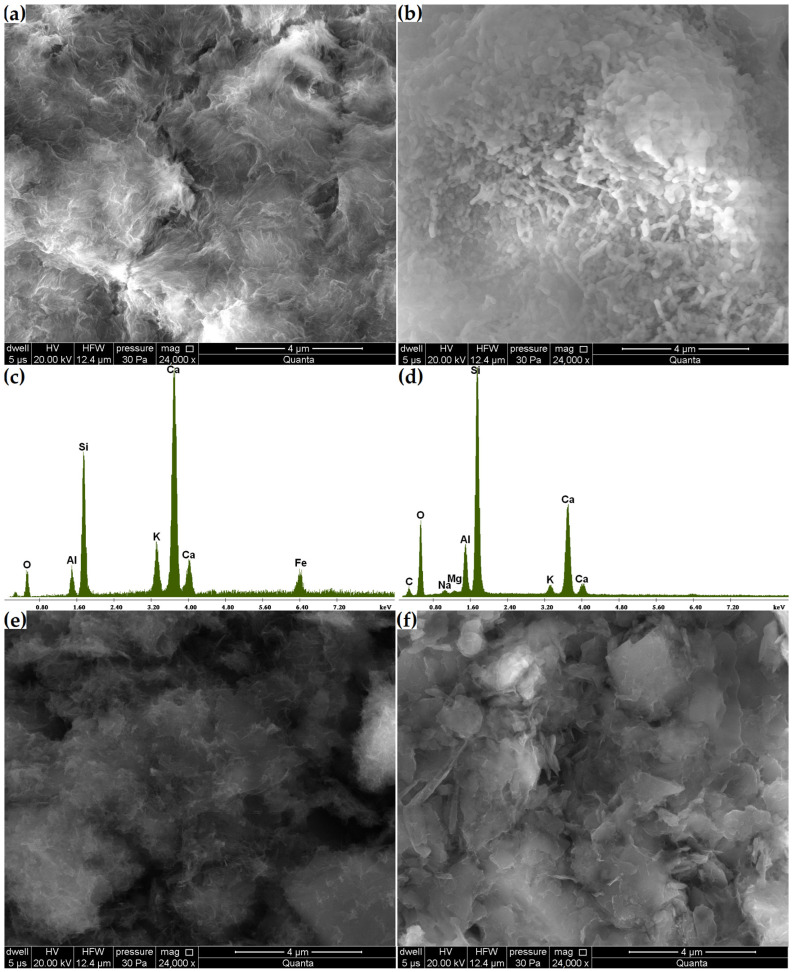
(**a**,**b**) Changes in the microstructure of the Z_c_ sample. (**c**,**d**) Elemental composition of Z_c_ areas in (**a**,**b**). Changes in the microstructures of samples (**e**) Z_n_ and (**f**) Z_k_.

Phase transitions affect microstructural changes. Compared to the original zeolite microstructure, in which monoclinic clinoptilolite crystals were found (Figure 4), in the Z_c_ sample, after 21 days, the formation of pozzolanic reaction products on its surface was observed, this being the C-S-H phase clearly visible at a magnification of 24,000× (Figure 10a,b). In addition to the fibrous amorphous product (Figure 10a), a more compact, rod-like product was also found (Figure 10b). The amorphous C-S-H phase corresponds to type III of Diamond’s morphological classification and often forms due to the hydration of cement with mineral additives [10]. Unlike its more compact form, the fibrous C-S-H phase is characterized by stronger indications from calcium and potassium and weaker indications from silicon (Figure 10c,d).

**Figure 11:**
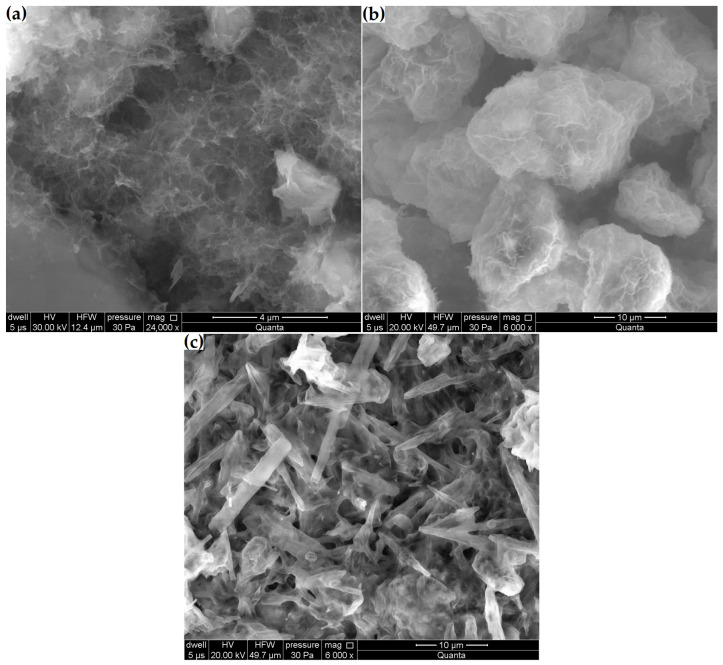
Microstructural changes in samples: (**a**) Z_cn_, (**b**) Z_ck_ and (**c**) Z_cks_.

Despite finding no compositional variations through the X-ray and thermal analyses, the grain surfaces of the Z_n_ and Z_k_ samples were covered with a layer of alkaline silica gel clearly visible at a magnification of 24,000×, as in the case of the C-S-H phase in Z_c_ (Figure 10e,f). This layer was so thin that the original contours of the underlying crystals could be observed. Fibrous reaction products were also found in the Z_cn_ sample (Figure 11a). A comparison of the products formed in the Z_n_ and Z_k_ samples showed that the sodium interaction resulted in a more amorphous product. This agrees with observations by Lu et al. [37] reported in their study on the alkali-aggregate reaction.

Changes in zeolite microstructure were clearly visible already at 6000× magnification in the samples with the highest decreases in clinoptilolite peak intensity and volume increases (Z_ck_ and Z_cks_). Figure 11b shows that the reaction products in the Z_ck_ sample densely coated the zeolite particles. In the Z_cks_ sample (Figure 11c), the reaction products filled the entire available space. In this sample, amorphous products were combined with needle-like crystalline products.

### 3.2. Microstructural Changes on the Surface of the Ground Section

**Figure 12:**
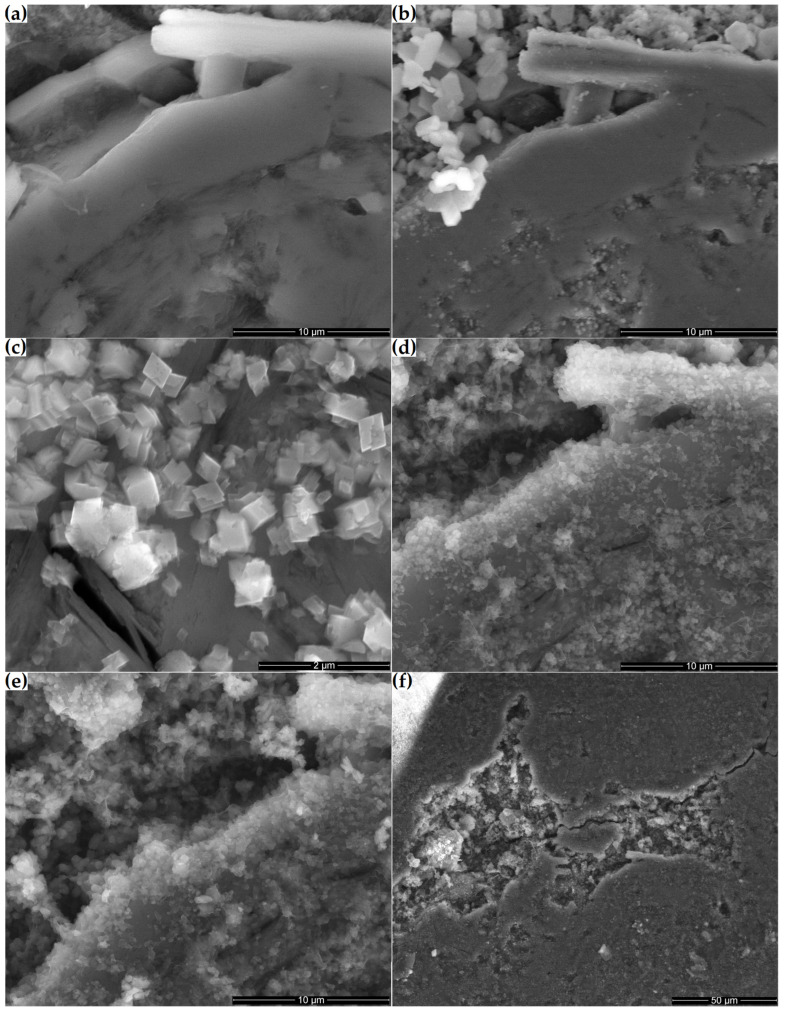
Microstructures of the zeolite ground section: (**a**) original sample and the Ca(OH)_2_-treated microstructure at (**b**) 1, (**c**) 7, (**d**) 14 and (**e**) 21 days; (**f**) pores and cracks in the 14-day sample.

Figure 12 shows the microstructural changes that occurred on the surface of a ground section placed in a supersaturated Ca(OH)_2_ solution over 21 days. Initially, the surface of the tested sample consisted primarily of a compact clinoptilolite mass with numerous small pores (Figure 12a). Clinoptilolite was also detected in the form of well-developed crystals in the macropores of the sample. After one day of storing the sample in the Ca(OH)_2_ solution, hexagonal portlandite crystals crystallized on its surface, mainly in the macropores (Figure 12b). After seven days, the hexagonal crystals were replaced by smaller orthorhombic crystals (Figure 12c). Their number increased with time, and the fibrous amorphous phase connecting them developed (Figure 12d,e). Over time, more compact products, such as those observed in Figure 10b, dominated the fibrous ones. Before the reaction products thus formed had time to cover the substrate, some cracks appeared on the surface of the 14-day and 21-day ground sections. Minor cracks occurred, among others, at the interface between the zeolite matrix and the well-crystallized clinoptilolite crystals present in the pores (Figure 12d). More extensive cracks also passed through the zeolite matrix, sometimes passing through the macropores (Figure 12f).

## 4. Discussion

Preliminary tests of Transcarpathian zeolite powder showed a material of 0–0.35 mm fraction. This size can be considered a coarse fraction for a mineral additive [6,8], as 0–0.05 mm zeolite is sold on the Polish market for the same purpose. It can thus be concluded that relatively large grains will negatively affect the chemical activity of the tested zeolite used as an additive [14,19,23]. This was proved by previous studies [38], which showed that only a zeolite fraction <0.125 mm undergoes pozzolanic reaction, beneficially affecting the properties of cement mortar.

Analysis of the phase composition of Transcarpathian zeolite revealed three potentially reactive phases. In addition to clinoptilolite, which is the primary constituent (67.07% by volume), there is also volcanic glass (12.40%) and pumice (2.50%). Furthermore, the volcanic glass may include reactive silica in the form of cristobalite, which was responsible for the significant intensity of the peak obtained in the diffraction pattern at 22.4°2θ [30,33]. Consequently, it can be concluded that 81.97% of the Transcarpathian zeolite volume can show some reactivity. This explains why, in further studies, despite the use of zeolite either as a powder with a small degree of fineness or in the form of a ground section, the effects confirming its entering into reactions with various solutions were quickly noticed.

For solutions containing sodium, potassium or calcium hydroxide, reactions occurred, resulting in the formation of an amorphous phase. It was noted that the interaction of hydroxides with monovalent cations was much weaker than that of Ca(OH)_2_. In the case of zeolite reaction with NaOH or KOH solutions, its manifestations could be detected most quickly with a scanning electron microscope at 24,000× magnification.

The changes occurring in the zeolite due to contact with a Ca(OH)_2_ solution were easily observed using various testing techniques. As a result of the pozzolanic reaction, the contents of the substrates necessary for its occurrence, i.e., portlandite and clinoptilolite, decreased over time in the samples. The results obtained from X-ray patterns and thermal analysis confirmed this process. Similar findings were reported by Ortega et al. [33] and Hou et al. [39]. Given that the clinoptilolite was the source of silica for the reactions, the decrease in the intensity of its peaks was analogous to those described by Martínez-Ramírez et al. [25] and Hamoudi et al. [40].

Over time, in addition to the substrates and inert materials included in the zeolite (e.g., quartz and feldspar), changes that may have indicated the formation of reaction products became noticeable in the diffractograms of some of the samples (Z_c_, Z_ck_ and Z_cks_). These include pronounced peaks that may have originated from hydrated calcium silicates and an increase in background within a 2θ range of 5–7°. The increase occurred in the region with the most intense peaks characteristic of some zeolite minerals, such as cowlesite (CaAl_2_Si_3_O_10_·5–6(H_2_O)) and mordenite ((Ca,Na_2_,K_2_)Al_2_Si_10_O_24_·7(H_2_O)) [30], which are hydrated aluminosilicates. Since all the elements needed for their formation were present in the studied systems, it cannot be ruled out that the amorphous phases formed in the samples could have structures similar to these zeolites. This was confirmed by the fact that the product of an alkali–silica reaction [15,16,17,18] or pozzolanic reaction [41] can crystallize in the form of zeolites.

In the Z_cks_ sample, which had SO_3_^2−^ ions in addition to Ca^2+^ and K^+^ ions as reaction products, ettringite (Ca_6_Al_2_(SO_4_)_3_(OH)_12_·26(H_2_O)) and, temporarily, syngenite (K_2_Ca(SO_4_)_2_·(H_2_O)) were also produced. Thus, this is a different process from that described by Ramachandran [11,38], in which monosulfate was the intermediate product. The syngenite was formed here without zeolite participation due to the reaction between Ca(OH)_2_ and K_2_SO_4_ in the solution. Nevertheless, zeolite, the only source of aluminium in the sample, must have been used to form ettringite. This confirms the theory that the pozzolanic reaction between zeolite and the Ca(OH)_2_ solution can result in the formation of ettringite in addition to the C-S-H phase [23,42]. The formation of ettringite and syngenite partially explains the Z_cks_ sample volume variation seen in Figure 2, as these are expansive phases [43].

The formed ettringite, whose characteristic needle-like crystals were present in the microstructure of the Z_cks_ sample after 21 days (Figure 11c), was also found to be an important element in the alkali-aggregate reaction [16,17,44].

When considering zeolite as an additive to Portland cement, it should also be noted that syngenite can be formed when clumping cement is exposed to humid air, leading to a rapid initial setting [10]. False cement setting can occur for this reason. However, Stark et al. [45] reported that syngenite, as an intermediate phase, can also occur during the regular hydration of cement made from clinker containing large amounts of potassium sulfate.

Microstructural transformations on the surface of the zeolite cross section showed that the C-S-H phase formed as a result of the pozzolanic reaction. This could eventually cover the entire surface of the zeolite containing clinoptilolite. There are several phases in this process:

- Contact of the solution containing Ca^2+^ with the zeolite, on the surface of which portlandite can crystallize (day 1 of the reaction—Figure 12b);
- Formation of fine, orthorhombic crystals, which may be hydrated calcium silicates (up to day 7 of the reaction—Figure 12c);
- Growth of the fibrous C-S-H phase around the orthorhombic crystals gradually covers the zeolite surface (after day 7—Figure 12d).

It was found that the produced gel forms of hydrated calcium silicates (observed via SEM) could contribute to a significant change in the volume of a sample. This phenomenon is promoted by the presence of K^+^ and SO_2_^3−^ ions and constrained by the presence of Na^+^. The interaction of SO_2_^3−^ ions can be linked to the effect of accelerating the pozzolanic reaction in the presence of gypsum, which is the carrier of these ions [23].

Changes in volume can be explained by the appearance of cracks on the surface of the zeolite ground section after 14 days of its being soaked in the Ca(OH)_2_ solution. Similar swelling of powdered rock samples was observed in the case of aggregates undergoing the alkali-aggregate reaction. In the study by Mitchell et al. [46], the swelling caused by NaOH action on carbonate rock powder at 20 °C was relatively small, as was the case with the zeolite discussed here.

The swelling of the zeolite powder samples was also correlated with the decrease in the intensity of the peaks of clinoptilolite, which was the source of reactive silica and microstructural changes. The interaction of Na^+^, K^+^, Ca^2+^ and SO_2_^3−^ ions coincides with the volume-change observations. The presence of Ca^2+^ ions is necessary for the process to occur; the K^+^ and SO_2_^3−^ ions intensify it, and Na^+^ ions limit the process. Changes in the intensity of the clinoptilolite and portlandite peaks in the presence of potassium were more substantial than those in the presence of sodium, as confirmed by the results of Hou et al. [39]. This may be because potassium ions can diffuse into reactive silica faster than sodium ions [47]. Cristobalite can also behave similarly, as the peak intensity at 22.4°2θ in Z_c_, Z_ck_ and Z_cks_ decreased significantly compared to the original zeolite diffraction pattern and was much smaller than in the diffraction patterns of other samples, e.g., Z_k_ (Figure 2 and Figure 9). Due to the volcanic origin of zeolite, as evidenced by the presence of volcanic glass, it can be concluded that cristobalite is present in the studied zeolite [48]. The precise interpretation of changes in this peak and in other peaks that may come from cristobalite, however, is problematic in the case of the tested samples due to overlaps with clinoptilolite peaks. For this reason, in future research, the problems of the chemical activity of clinoptilolite and cristobalite should be investigated separately.

The loss of the second substrate of the pozzolanic reaction, namely, portlandite, is not clearly related to the swelling of the sample. Compared with Z_ck_, a faster and more significant decrease in portlandite content was observed in the Z_cn_ sample diffractograms and thermal analysis results. The portlandite content changed the least in the presence of potassium (Z_ck_). However, when this system was modified by introducing sulfur (Z_cks_), both clinoptilolite and portlandite showed the most significant decreases in content over the 180-day study. At 180 days, portlandite reacted entirely in the Z_cks_ sample. The increased consumption of clinoptilolite and portlandite in this sample may be related to the pozzolanic reaction and the second reaction resulting in the formation of ettringite.

Reduced portlandite content is also associated with fluctuations in the content of carbonates. They are formed due to the carbonation of portlandite in the form of CaCO_3_, which consumes the available Ca(OH)_2_ [34]. This process could be observed during the thermal analysis of the Z_c_ sample, in which the carbonate content increased over time at the expense of portlandite. However, in samples with other hydroxides, in addition to Ca(OH)_2_, a progressive loss of calcium carbonate and hydroxide took place over time. This observation converged with the findings of Martínez-Ramírez et al. [25] on the effects of Ca(OH)_2_ on the clinoptilolite-like zeolite heulandite. The so-called “common ion effect” which disturbs the balance between Ca(OH)_2_ and CaCO_3_ [49,50], which can be converted to portlandite and consumed during the reaction with clinoptilolite, could be a factor here.

## 5. Conclusions

As a result of the tests performed, it was possible to describe how selected ions, for example, those present in concrete pore solution, interact with zeolite rock primarily composed of clinoptilolite.

The main conclusions from this study are as follows:

- Due to the high content of reactive phases, particularly clinoptilolite, Transcarpathian zeolite can react with solutions containing Na^+^, K^+^ and Ca^2+^ ions. Various forms of gel resulting from the reactions cover the surface of the zeolite.
- The action of the solution containing dissolved calcium hydroxide on the zeolite leads to a pozzolanic reaction. The decrease in the content of clinoptilolite and portlandite in the tested samples evidences its course.
- The effects of zeolite reaction with the calcium hydroxide solution largely depend on the presence of other ions (e.g., Na^+^, K^+^ and SO_2_^3−^). These ions affect the rate of reactions and the observed volume changes that can form gel-like products.
- In the presence of potassium hydroxide, zeolite reacts faster in the pozzolanic reaction and produces more gel products, but portlandite reacts faster in the presence of sodium hydroxide. This phenomenon can be clarified by examining the composition of the resulting products in more detail.
- In addition to the pozzolanic reaction, calcium hydroxide can participate in other reactions simultaneously. As a result, in the presence of Ca(OH)_2_ and SO_2_^3−^ ions in the solution, a parallel formation of ettringite and syngenite as an intermediate product can occur, thus contributing to a higher degree of clinoptilolite and portlandite conversion. However, the course of this reaction and its impact on the formation of gel phases require further research.
- The demonstrated reactivity of clinoptilolite with Na^+^ and K^+^ ions can cause large zeolite grains or agglomerates to potentially react in the alkali–silica reaction.

Further studies should consider the effect of exchangeable cations present in clinoptilolite on the chemical activity of the zeolite. This is relevant, as the same ions, K^+^, Na^+^ and Ca^2+^ [1,2], from the external source are the focus of this article. In future research, the chemical activity of the Transcarpathian zeolite should be investigated not in model systems, but in real systems. Such research can be carried out gradually; first, by replacing the model pore solution with a real one obtained from concrete [51], and then in a cement paste environment and concrete. It would also be necessary to confirm with standard studies whether zeolites as aggregates can enter into the alkali–silica reaction and what effects this would have.

## Figures and Tables

**Table 1:** Chemical composition (oxide content) of the zeolite studied (wt.%).

Oxide	SiO_2_	Al_2_O_3_	Fe_2_O_3_	CaO	MgO	K_2_O	Na_2_O	TiO_2_	MnO	P_2_O_5_	Na_2_O_eq_ *
Zeolite	67.07	12.40	0.90	2.09	0.72	2.80	2.05	0.19	0.04	0.014	3.89

* Na_2_O_eq_ = Na_2_O + 0.658K_2_O.

**Table 2:** Mineral composition of the zeolite.

Mineral	Content (vol.%)
Clinoptilolite	65
Volcanic glass	14
Quartz	6
Plagioclase	1
Sanidine	3
Biotite	2
Pumice	6
Iron hydroxides	3

**Table 3:** Compositions of powder samples for zeolite activity testing (g).

Sample	Zeolite	Ca(OH)_2_	Water	1M NaOH Solution	1M KOH Solution	0.5M K_2_SO_4_ Solution
Z_c_	15	5	66.5	-	-	-
Z_n_	15	-	-	66.5	-	-
Z_k_	15	-	-	-	66.5	-
Z_cn_	15	5	-	66.5	-	-
Z_ck_	15	5	-	-	66.5	-
Z_cks_	15	5	-	-	-	66.5

**Table 4:** Thermal analysis results for the Transcarpathian zeolite powder samples (%).

Sample	H_2_O from Hydrate	H_2_O from Ca(OH)_2_	Ca(OH)_2_	CO_2_	CaCO_3_	Ca(OH)_2_ *	Increase in Ca(OH)_2_ * between Days 7 and 180
Z_n_7	10.11	-	-	-	-	-	-
Z_n_180	10.83	-	-	-	-	-	
Z_k_7	10.83	-	-	-	-	-	-
Z_k_180	11.61	-	-	-	-	-	
Z_c_7	6.67	5.45	22.43	0.88	2.01	23.92	−77.29%
Z_c_180	11.99	0.60	2.49	1.75	3.98	5.43	
Z_cn_7	7.73	1.70	7.00	7.81	17.76	20.15	−48.54%
Z_cn_180	13.10	0.71	2.94	4.41	10.03	10.37	
Z_ck_7	10.68	1.94	8.00	6.95	15.81	19.70	−13.65%
Z_ck_180	12.69	1.58	6.49	6.25	14.21	17.02	
Z_cks_7	9.98	1.27	5.24	3.10	7.06	10.47	−86.47%
Z_cks_180	11.63	0.00	0.00	0.84	1.91	1.42	

* Ca(OH)_2_ contents, including carbonated amounts.

## Data Availability

Not applicable.
